# Supplementary figures and images for: Molecular evidence for sex reversal in wild populations of green frogs (Rana clamitans)
Source: PeerJ. 2019 Feb 8;7:e6449. doi: 10.7717/peerj.6449 (PMC6369831; doi:10.7717/peerj.6449)

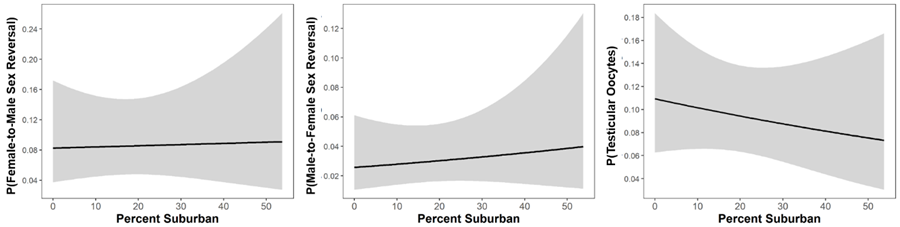

Supplement: Supplemental Information 1 — Relationships between frequencies of female-to-male sex reversal, male-to-female sex reversal, and intersex (probability of a male with testicular oocytes) with the Percent of Suburban land cover surrounding ponds. Sex reversal and intersex frequencies showed no significant relationship with suburban land cover. [file peerj-07-6449-s001.png]

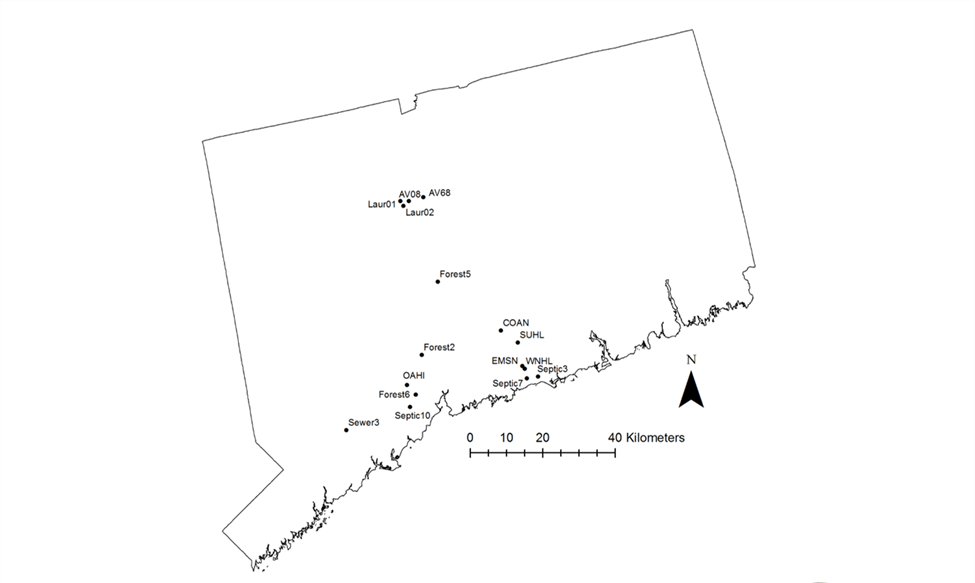

Supplement: Supplemental Information 2 — Entirely forested ponds are dispersed across the spatial distribution of ponds here as are ponds with varying degrees of suburban land cover. Pond names correspond to those in Table S1 and the degree of suburban land cover surrounding each pond can also be found in Table S1. [file peerj-07-6449-s002.png]

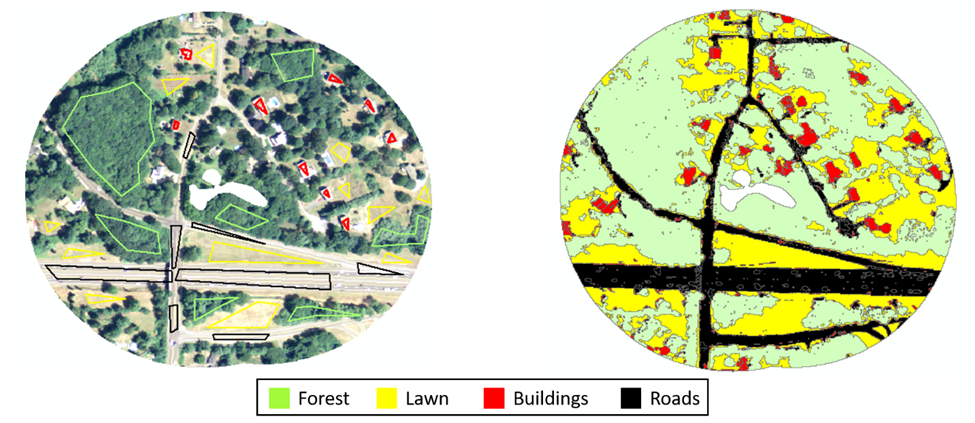

Supplement: Supplemental Information 3 — On the left is a 4-band, 1-m resolution orthoimage constrained to a 200-m buffer surrounding a suburban pond (center). Different color polygons on the image represent forest (green), lawn grasses (yellow), buildings (red), and roads (black) used to train GIS algorithm to different clusters of wavelengths. Right is the associated image at the completion of the classification. We performed the supervised classification simultaneously on all 16 ponds, using representative training polygons of all available land cover types around each pond to train the model. [file peerj-07-6449-s003.png]

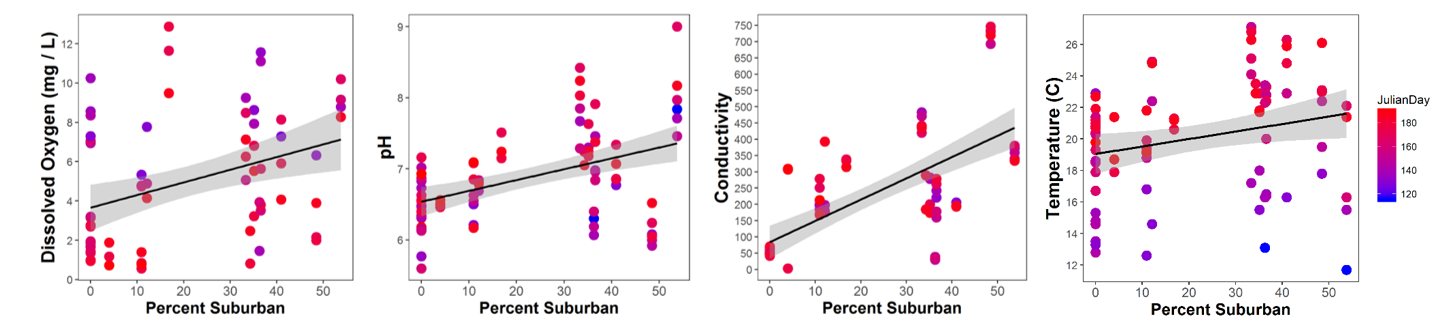

Supplement: Supplemental Information 4 — Dissolved oxygen decreased across the season but increased with suburban land use. Water pH increased across the season and with suburbanization. Specific conductance (i.e., conductivity) was predominantly invariable across sampling dates within a pond but was strongly correlated with suburban land use. Water temperature increased across the sampling season and was also slightly positively correlated with suburban land use. [file peerj-07-6449-s004.png]
